# Effects of an intervention to improve sexual and reproductive health on level and predictors of awareness and knowledge of condoms and dual protection amongst adolescents in Nigeria

**DOI:** 10.1186/s40834-024-00270-2

**Published:** 2024-03-04

**Authors:** Chibuike Agu, Ifeyinwa Chizoba Akamike, Ifunanya Agu, Ozioma Agu, Godstime O. Eigbiremolen, Chinyere Ojiugo Mbachu, Obinna Onwujekwe

**Affiliations:** 1 Health Policy Research Group, College of Medicine, University of Nigeria, Ituku Ozalla, Enugu State Nigeria; 2 Department of Community Medicine, College of Medicine, University of Nigeria, Ituku Ozalla, Enugu State Nigeria; 3 Department of Community Medicine, Alex Ekwueme Federal University Teaching Hospital, Abakaliki, Ebonyi State Nigeria; 4 Department of Economics, University of Nigeria, Nsukka, Enugu State Nigeria

**Keywords:** Adolescents, Knowledge, Pregnancy prevention methods, Condom use, dual protection, Predictors

## Abstract

**Background:**

Adolescents need both information about sexual behaviours and potential risks in order to make the right choices. This study compared adolescents’ level of awareness and predictors of knowledge of condoms and dual protection where a multi-component sexual and reproductive health (SRH) intervention was implemented and in communities where the intervention was not implemented, so as to understand the effect of the intervention on awareness and knowledge and also identify predictors of knowledge of contraception methods.

**Methods:**

The study was an intervention study that was undertaken in six local government areas (three rural and three urban LGAs) in Ebonyi state, southeast Nigeria. Data were collected from 855 adolescent boys and girls, using a pre-tested interviewer administered questionnaire. Systematic random sampling technique was used to select eligible households from which adolescents were interviewed Analysis of data was carried out using bivariate and multivariate linear regression analyses. The level of statistical significance was determined by a p value of < 0.05.

**Results:**

The level of awareness of condoms and dual protection was similar in the intervention and non-intervention communities. However, the predictors of knowledge about condoms and dual protection were different between the intervention and non-intervention groups. The multivariate linear regression showed that the higher the level of education, the higher the level of awareness of contraception methods among adolescents (*p* < 0.05). Likewise, increasing age by one year and working for pay increased the awareness of condoms and dual protection(*p* < 0.05).

**Conclusion:**

There was no difference in the level of awareness of pregnancy prevention methods, knowledge of condoms and dual protection in both arms of the study. Higher level of education, increasing age, and working for pay are factors associated with awareness of condom and dual protection. These factors should be prioritized for effective Adolescent sexual and reproductive health (ASRH) programming.

## Introduction

In order to ensure that the sexual and reproductive health (SRH) and wellbeing of adolescents are maintained, adolescents need access to age-appropriate comprehensive sexuality education that will enable them to develop life skills. They also need access to acceptable, equitable, appropriate and effective SRH services that are provided in safe and supportive environments [1].

Adolescents need basic information about the reproductive system as well as more advanced knowledge of sexual behaviours and potential risks so as to make the right decisions that protect against risky sexual behaviors [2]. Although, comprehensive education is being promoted in formal education settings (such as secondary schools) in Nigeria, this approach systematically excludes adolescents who are out-of-school and therefore more vulnerable because they have limited access to information.

Poor SRH outcomes among adolescents contributes significantly to the global burden of sexual ill health [3]. Sexual and reproductive health of adolescents is of particular importance because it focuses on the physical and emotional wellbeing of adolescents and enables them prevent unwanted pregnancy, unsafe abortion, STIs (including HIV/AIDS), and sexual violence and coercion [4]. The high burden of poor SRH among adolescents has been linked to their level of knowledge of SRH issues [8]. Many studies have reported poor knowledge of SRH issues among adolescents in SSA [2, 5, 6]. A study which analyzed data from eight health and demographic surveillances sites (HDSS) in six sub-Saharan African (SSA) countries, including Nigeria, Burkina Faso, Ethiopia, Ghana, Tanzania, and Uganda, reported that many adolescents in the sub-region lack knowledge of SRH [2]. Also, in Lao People’s Democratic Republic, lack of knowledge of SRH issues and low level of awareness of services were identified as the main barriers to accessing SRH services among adolescents [7].

Evidence has shown that education has a role to play in improving awareness about different aspects of life including health and it is a fundamental social determinant of health [8]. Adolescent sexual and reproductive health ASRH) has been included in school curriculums and it is therefore expected that those who have some level of education should be informed about the topic. Hence, it is hoped that interventions that could increase the level of awareness and knowledge of adolescents about contraceptives will positively affect their view points and improve their appropriate demand for such goods and services. Based on this premise, a suite of interventions was co-produced with community members by a research team and implemented in Ebonyi state, southeast Nigeria. This intervention comprised of group awareness campaigns on adolescent SRH in communities. The community campaigns made use of trained school teachers, peer educators and health workers who were already trained on adolescent SRH in the communities to deliver information to smaller groups of the target audiences on adolescent sexual and reproductive health. Information and education communication (IEC) materials (handbills and posters) were prepared and displayed in strategic places in the communities, and distributed to households.

Condom is a barrier method of contraception which in addition to its function in pregnancy prevention also protects against sexually transmitted infections (STIs) including Human immunodeficiency virus (HIV) infection. This dual function provided by the condom is known as dual protection. The focus on condom and dual protection in this study is due to the fact that adolescents may less likely use other methods of pregnancy prevention because of the notion that other methods should be left for married couples. Again, it’s been shown that condom is the preferred method of contraception among adolescents and young adults in developing countries [9].

This paper presents information on the effectiveness of the intervention on the level of awareness of various pregnancy prevention methods and the knowledge about condom and dual protection among adolescents in the intervention and non-intervention communities as well as identify predictors of awareness of pregnancy prevention methods, knowledge of condom and dual protection. The evidence provided by this study would be useful to policymakers, program managers and service providers in designing, planning and implementing appropriate sexual and reproductive health programmes for adolescents.

## Methods

### Study area

This was an intervention study that was undertaken in six communities where the intervention on SRH were implemented for adolescents and six communities where the interventions were not implemented. These communities were selected from six local government areas (LGAs) in Ebonyi State, southeast Nigeria.The state is located on latitude: 6° 15’ 18” N, longitude: 8° 05’ 55” E, and shares a border with Benue State to the north, Enugu State to the west, Imo and Abia States to the south and Cross River State to the east.

The prevalence of teenage pregnancy is high in Ebonyi state in addition to a high unmet need for contraceptives among adolescents and young people. These six LGAs were listed by stakeholders as having the highest unwanted teenage pregnancy rates in the State.

The communities where ASRH intervention was carried out include Agbaja- Nnuhu in Abakaliki LGA, Nwofe in Izzi LGA, Abina-Ikwo in Ikwo LGA, Onueke (Amuzu) in Ezza South LGA, Ebunwana in Afikpo South LGA and Okposi-Okwu in Ohaozara LGA. The non-intervention communities were Amagu Unuhu in Abakaliki LGA, Ndieze in Izzi LGA, Ekpelu in Ikwo LGA, Okoffia in Ezza South LGA, Oso Edda in Afikpo South LGA and Ugwulangwu in Ohaozara LGA.

### The intervention

The baseline findings on awareness and use of contraceptive amongst adolescents have been published previously before the intervention was implemented [10, 11]. The intervention comprised of group awareness campaigns on adolescent SRH in communities. Trained school teachers, peer educators and health workers who were already trained on adolescent SRH in the communities to deliver information to smaller groups of the target audiences on adolescent sexual and reproductive health were used in the implementation of the campaigns.

Information and education communication (IEC) materials (handbills and posters) were prepared and displayed in strategic places in the communities, and distributed to households. The target audiences included adolescent boys and girls, parents and guardians of adolescents, and community leaders. A total of five interactive campaign sessions were held with a maximum of 20 participants in each community.

Three (3) separate sessions were organized for adolescent boys and girls, one (1) session was organized for parents and guardians of adolescents, and another session was organized for community leaders/influencers (including religious leaders, traditional leaders, village heads, youth leaders, women leaders, men leaders and representatives of community vigilante).

The sessions with adolescents and adults were facilitated by boundary partners comprising trained school teachers, peer educators, and adolescent health focal officers of the LGA. Topics covered included, the sexual and reproductive health and rights of adolescents, sexual abstinence, contraception and prevention of unwanted pregnancy, life-skills for preventing rape, sexual exploitation and abuse, reporting of rape, sources of SRH information and services for adolescents, and effective parent-child communication of SRH matters. The intervention lasted for two years between October 2019 and August 2021, and the evaluation of the intervention was carried out six months after the intervention.

### Study population, and sampling

The study population consisted of adolescents aged 13 to 18 years who resided in selected households in the communities, and parents/caregivers of these adolescents. Adolescents aged 13–17 years whose parents/caregivers were not available to consent at the time of the survey were excluded from the interview. A total of 855 adolescents (intervention 413, non-intervention 442) were interviewed from 317 households across the twelve communities.

Systematic random sampling technique was used to select eligible households from which adolescents were interviewed. The nearest public facility (primary health center, school, church or town hall) from the main entrance to the community was used as the starting point from which recruitment of households began. Households were selected through a random walk from the starting point. The next consecutive household was then selected from the recruited ones until the desired sample size was reached. All eligible adolescents residing in the selected households were invited to participate in the study. All visitors or guests were excluded. One repeat visit was made to each household to recruit adolescents who were absent during the first visit.

### Data collection

The data collection instrument was adapted from the WHO illustrative questionnaire for interview-surveys with young people [12]. The structured questionnaire was pre-tested in one rural and one urban ASRH intervention communities among target population. The questions focused on adolescents’ awareness and knowledge about SRH, and multiple answers were allowed.

### Data analyses

Data analysis was carried out using STATA software. Frequencies and proportions were calculated. Chi square test was carried out to determine the differences between intervention and non-intervention arms.

In order to determine the predictors of knowledge regarding condom use and dual protection among adolescents in community, descriptive statistics and multivariate linear regression analysis were used. Our regression model allowed us to take our analysis further by isolating specific predictors of knowledge regarding condom use and dual protection, while taking into account variations in individual socioeconomic and socio-demographic characteristics under a regression framework. The multivariate regression model can be specified parsimoniously as:

1 $$ {Y}_{i}={\beta }_{0}+{\beta }_{1}{X}_{i}+{\mu }_{i}$$

Where $$ {Y}_{i}$$ represents the outcome variable for individual *i*. The outcomes of interest include awareness of pregnancy prevention methods among adolescents (measured by 10 questions shown on Table 1), knowledge of condom use among adolescents (measured by 13 questions presented on Table 2), and knowledge of dual protection among adolescents (measured by one question presented on Table 3). $$ {X}_{i}$$ is a vector of control variables for individual *i*, which it includes gender, level of education, area of residence (whether urban or rural), age, whether an individual works for pay or not, and whether an individual belongs to the intervention group or not. The error term, $$ {\mu }_{i}$$, is taken to be normally distributed.

**Table 1:** Awareness of pregnancy prevention methods

Variables	Intervention group Frequency (%) *n* = 413	Non-Intervention group Frequency (%)*n* = 442	x^2^ (*p*value)
**Methods of preventing pregnancy respondents had heard about**			
Abstinence	324 (78.45)	326 (73.76)	2.582 (0.108)
Oral contraceptive pill	68 (16.46)	71 (16.06)	0.025 (0.874)
Injectable contraceptives	48 (11.62)	35 (7.92)	3.341 (0.068)
Male condom	258 (62.47)	279 (63.12)	0.0389 (0.844)
Female condom	112 (27.12)	132 (29.86)	0.789 (0.374)
Withdrawal method	38 (9.20)	36 (8.14)	0.301 (0.583)
Calendar/Rhythm method	18 (4.36)	23 (5.20)	0.334 (0.563)
Basal body temperature	2 (0.48)	1 (0.23)	0.407 (0.524)
Vaginal mucus inspection	2 (0.48)	6 (1.36)	1.756 (0.185)
Emergency pill	9 (2.18)	13 (2.94)	0.495 (0.482)
**Composite score for Awareness: Mean (SD)**	2.128 (1.411)	2.086 (1.384)	-0.4430** (0.660)

* Significant (*p* < 0.05)

**Table 2:** Knowledge of condom***

Variables	Intervention group Frequency (%) *n* = 413	Non-Intervention group Frequency (%) *n* = 442	x^2^ (*p*value)
Condoms are an effective method of preventing pregnancy	231 (55.93)	260 (58.82)	0.730 (0.393)
Condoms can be used more than once	39 (9.44)	39 (8.82)	0.099 (0.753)
A girl can suggest to her boyfriend that he use a condom	261 (63.20)	277 (62.67)	0.025 (0.873)
A boy can suggest to his girlfriend that he use a condom	276 (66.83)	300 (67.87)	0.106 (0.745)
Condoms are an effective way of protecting against STIs (e.g. HIV/AIDS)	261 (63.20)	312 (70.59)	5.278 (0.022)*
Condoms are suitable for casual relationships	179 (43.34)	221 (50.00)	3.802 (0.051)
Condoms are suitable for steady, loving relationships	185 (44.79)	205 (46.38)	0.217 (0.642)
It would be too embarrassing for someone like me to buy or obtain condoms	263 (63.68)	308 (69.68)	3.468 (0.063)
If a girl suggested using condoms to her partner, it would mean that she didn’t trust him	160 (38.74)	162 (36.65)	0.3970 (0.529)
Condoms reduce sexual pleasure	94 (22.76)	89 (20.14)	0.874 (0.350)
Condoms can slip off the man and disappear inside the woman’s body	114 (27.60)	126 (28.51)	0.086 (0.769)
If unmarried couples want to have sexual intercourse before marriage, they should use condoms	206 (49.88)	220 (49.77)	0.0009 (0.975)
Condoms are an effective way of protecting against sexually transmitted diseases	280 (67.80)	322 (72.85)	2.6176 (0.106)
**Composites Score of Knowledge on condom: Mean(SD)**	6.172 (3.510)	6.427 (3.296)	1.099** (0.274)

*statistical significance **t test *** multiple responses allowed

**Table 3:** Most suitable contraceptive method for adolescents and knowledge about dual protection (methods of preventing pregnancy that also prevent STIs)

Variables	Intervention group Frequency (%) *n* = 413	Non-Intervention group Frequency (%) *n* = 442	x^2^ (*p* value)/T-test
**The most suitable contraceptive method for adolescent**			
Abstinence	308 (69.68)	291 (70.06)	1.8361(0.934)
Male condom	88 (19.91)	81 (19.61)	
Oral contraceptive			
Calendar/Rhythm method	12 (2.71)	8 (1.94)	
Others			
**Knowledge of methods of preventing pregnancy that also prevent STIs			
Abstinence	309 (74.82)	313 (70.81)	1.727 (0.189)
Male condom	188 (45.52)	215 (48.64)	0.835 (0.361)
Female condom	70 (16.95)	88 (19.91)	1.242 (0.265)
**Composite Score of Knowledge on dual protection: Mean(SD)**	1.373 (0.770)	1.394 (0.767)	0.395* (0.690)

*t test ** multiple responses allowed

### Ethical considerations

Ethical approval for the study was obtained from the Health Research Ethics Committee of University of Nigeria Teaching Hospital Enugu and the Research and Ethics Committee of Ebonyi State Ministry of Health. Ethical approval was secured from both committees before entry into the study site. Informed written consent was obtained from parents/guardians of adolescents aged 13 to 17 years who participated in the survey. Also, a written assent was obtained from adolescents aged 13 to 17 years and older adolescents aged 18 years gave informed consent for the study.

## Results

The baseline findings on awareness and use of contraceptives amongst adolescents have been published previously. Table 4 shows that the two arms of the study were comparable in their socio-demographic characteristics (*p* > 0.05). Many of the respondents were females and in junior secondary school. About 50% of the adolescents reside in the rural area in both intervention and non-intervention groups.

**Table 4:** Socio-demographic characteristics of respondents

Variables (*N* = 855)	Intervention group Frequency (%) *n* = 413	Non-intervention group Frequency (%) *n* = 442	Ttest-value
**Gender**			
Female	238(57.63)	253(57.24)	-0.1143
Male	175(42.37)	189(42.76)	
**Level of education completed**			
Senior	115(29.26)	105 (24.03)	-1.7070
Junior	278(70.74)	332 (75.97)	
**Place of residence**			
Urban	198(47.94)	217(49.10)	0.3368
Rural	215(52.06)	225(50.90)	
**Age in single years**			
13	81(19.61)	84(19.00)	0.0824
14	60(14.53)	64(14.48)	
15	67(16.22)	77(17.42)	
16	64(15.50)	72(16.29)	
17	71(17.19)	63(14.25)	
18	70(16.95)	82(18.55)	
***Mean age (std dev)***	15.46 (1.75)	15.47(1.75)	15.47 (0.934) ^
**Working for pay**			
Yes	79(19.13)	80(18.10)	-0.3859
No	334(80.87)	362(81.90)	

^Student’s t-test, * significant (*p* < 0.05)

Table 1 shows that abstinence and male condom were the major methods of pregnancy prevention that respondents were aware of. About one third were aware of female condom as a method of preventing pregnancy. There was no significant statistical differences in awareness of pregnancy prevention methods in the two groups.

Table 2 shows that the mean score for knowledge of condom was 6.172 ± 3.510 and 6.427 ± 3.296 (in intervention and non-intervention arms respectively). There was no statistically significant difference in the responses from both arms of the study, except for the question on effectiveness of condom in protecting against STIs such as HIV/AIDS which had higher proportion of respondents (70.59%) in non-intervention arm answering “yes” compared to 63.20% in the intervention arm.

Table 3 shows that the majority of the respondents indicated that abstinence was the most suitable contraceptive method for adolescents, and the proportions were comparable in both intervention and non-intervention communities, (χ^2^ = 1.8361, *p* = 0.934). Also, the majority and similar proportions of the respondents in both arms (74.82% and 70.81 in intervention and non-intervention arms respectively) reported that abstinence is a method of dual protection while less than 50% listed use of male condom as a method of dual protection. Only about one fifth in both arms knew that use of female condom is a method of dual protection. All the respondents knew that other methods including oral contraceptive pills, injectables, withdrawal method, calendar method, basal body temperature method, vaginal mucus inspection and emergency pills are not used for dual protection. The mean knowledge score about dual protection in both arms is similar.

Table 5 shows predictors of awareness of pregnancy prevention methods and knowledge of condom and dual protection among adolescents in communities, which were education, age, and working for pay. The table shows that higher education significantly increases the likelihood of having awareness of pregnancy prevention methods by 21.0%. Likewise, increasing age by 1 year increases the likelihood of awareness of pregnancy prevention methods by 7.1%. Working for pay significantly increases the likelihood of having awareness of pregnancy prevention methods by 14.3%. Being in the intervention group increases the likelihood of having awareness of pregnancy prevention methods among adolescents in communities by 12.4% but the difference was not statistically significant. The only predictor of knowledge of condom among adolescents in communities was age. Increasing age by 1 year increases the likelihood of knowledge of condom by 12.3%.

**Table 5:** Multiple linear regression analysis for predictors of awareness of pregnancy prevention methods and knowledge of condom and dual protection

Socio-demographic characteristics	Pregnancy PM	Condom	Dual protection
	Coefficient (T-test)	Coefficient (T-test)	Coefficient (T-test)
**Gender (female)**	0.043 (1.12)	-0.037 (-0.89)	-0.058 (-1.87)
**Level in school (Senior Secondary)**	0.218 (4.45*)	-0.049 (-0.94)	0.137 (3.50*)
**Location of residence (urban)**	0.363 (-0.91)	-0.041 (-0.98)	-0.158 (-4.83*)
**Age(Increasing age)**	0.071 (5.51*)	0.123 (8.80*)	0.019 (1.85)
**Working for pay**	0.143 (2.81*)	0.047 (0.84)	0.091 (2.24*)
**Intervention (treatment)**	0.014 **(0.38)**	**-**	-0.011 (-0.37)

*Statistical significance (t > 1.96; *p* < 0.05) PM (Prevention method)

The predictors of knowledge of dual protection among adolescents in communities were education, working for pay and residence. Higher education significantly increases the likelihood of having knowledge of dual protection by 13.7%. Working for pay significantly increases the likelihood of having knowledge of dual protection by 9.1%. Living in the urban area, significantly decreases the likelihood of having knowledge of dual protection by 15.8%.

## Discussion

This study compared the knowledge about pregnancy prevention, condom, and dual protection methods among intervention and non-intervention communities after implementation of community wide interventions. The finding that there was no statistically significant difference in awareness about pregnancy prevention, knowledge about condom, and dual protection in both arms of the study, except that higher proportion of respondents in non-intervention communities agreed that condom is effective in protecting against STIs such as HIV/AIDS, is surprising considering the fact that only those in the intervention arm received the interventions and would be expected to have better knowledge than the non-intervention communities.

The finding that abstinence was the major method of pregnancy prevention the respondents were aware of, followed by male condom in both intervention and non-intervention arm have been reported in a previous study [13]. These findings reflect the fact that adolescents still have value for abstinence however, knowledge does not always translate to practice. Their responses may have been influenced by the culturally accepted notion of abstinence for unmarried individuals. It is therefore necessary to continue the provision of comprehensive and correct information on sexual and reproductive health to adolescents in communities.

The finding on the predictors of knowledge that showed that receiving the intervention had no effect on awareness of pregnancy prevention methods and on the knowledge of condom and dual protection aligns with a study carried out in Laos [14]. Acquiring information early is crucial for the adolescents because they are able to make informed choices when faced with certain issues. A major concern is that the society, including religious groups is mostly in support of abstinence and therefore frowns at the use of condoms or contraceptives by adolescents.

An inference from the findings that senior secondary education and working for pay significantly increase the likelihood of having knowledge of dual protection buttresses the importance of education in improving knowledge and highlights the peculiarities of adolescents who are involved in paid jobs. Consequently, adolescent sexual and reproductive health interventions targeting this group should be equally emphasized in communities and not limited to school settings. The finding that living in the urban area significantly decreases the likelihood of having knowledge of dual protection may be explained by the fact that the rural communities have more exposures with community health campaigns and most health programs are being implemented in rural areas. On the contrary, a study carried out in India reported that urban residents had better knowledge of SRH [15]. However, the Indian study was carried out among older youths; this may be the reason for the difference.

Knowledge about dual protection is crucial because HIV-related deaths are not decreasing among adolescents and the levels of other sexually transmitted infections are high and growing among them [16]. However, it is surprising that a higher percentage of respondents in the non-intervention group had a better knowledge of dual protection.The finding, Increasing age by 1 year increases the likelihood of awareness of SRHis not unexpected since older adolescents are exposed to certain information by virtue of experiences they have had as well as through interaction with peers. The effect of increasing age was also reported in a similar study carried out in Laos [14].

Working for pay significantly increases the likelihood of having awareness of SRH. Adolescents who are engaged in paid jobs may be more likely to have more awareness by virtue of their exposures at work. This highlights the need for targeted and continuous ASRH education to ensure that the right information gets to these adolescents at the appropriate stage of their development. Adolescents who are not in school are likely to get involved in paid jobs at an early stage in their lives and therefore must be equipped with the necessary information for their wellbeing. A study reported that adolescents who worked more than 120 h a month were significantly more likely than non-working adolescents to experience first intercourse early[17].

This study further revealed that majority of adolescents indicated abstinence as the most suitable contraceptive method for adolescents. This finding can be explained by the fact that abstinence aligns with practices in many African communities, where most traditions encourage chastity. In addition, some communities have measures in place to promote and enforce sexual abstinence [18]. Such traditions are to be encouraged and sustained considering the benefits it has on adolescent’s sexual behaviours.While this study had a large sample size, it was based on self-reports and thus, there may have been information and social desirability bias. However it addressed a sensitive issue and provides evidence for decision making as regards adolescent sexual and reproductive health.

## Conclusions

There was no difference in the level of awareness of pregnancy prevention methods, knowledge of condoms and dual protection in both arms of the study. Higher level of education, increasing age, and working for pay are factors associated with awareness of condom and dual protection. These factors should be prioritized in the design, planning, and implementation of appropriate and effective ASRH programmes.

## Data Availability

The dataset for the study can be obtained from the corresponding author upon reasonable request.

## References

[1] World Health Organisation. Adolescent Health. Available from: https://www.who.int/health-topics/adolescent-health#tab=tab. Accessed January 2022.

[2] Finlay JE, Assefa N, Mwanyika-Sando M, Dessie Y, Harling G, Njau T (2020). Sexual and reproductive health knowledge among adolescents in eight sites across sub-saharan Africa. Trop Med Int Heal.

[3] Morris JL, Rushwan H. Adolescent sexual and reproductive health: The global challenges. Int J Gynecol Obstet [Internet]. 2015;131(2015):S40–2. 10.1016/j.ijgo.2015.02.006. 10.1016/j.ijgo.2015.02.006 26433504

[4] The Open University. Introduction to Adolescent and Youth Reproductive Health (AYRH). 2022.

[5] Abiodun O, Ani F, Sotunsa O, Olu-Abiodun O. Sexual and Reproductive Health knowledge and service utilization among In-school rural adolescents in Nigeria. J AIDS Clin Res. 2016;7(6). 10.4172/2155-6113.1000576.

[6] Adeokun L, Ricketts O, Ajuwon A, Ladipo O (2009). Sexual and reproductive health knowledge, behaviour and education needs of in-school adolescents in northern Nigeria. Afr J Reprod Heal.

[7] Thongmixay S, Essink DR, DeGreeuw T, Vongxay V, JEWB VS (2019). Perceived barriers in accessing sexual and reproductive health services for youth in Lao people’s Democratic Republic. PLoS ONE.

[8] Hahn RA, Truman BI (2015). Education Improves Public Health and Promotes Health Equity. Int J Heal Serv.

[9] Barchi F, Ntshebe O, Apps H, Ramaphane P (2022). Contraceptive literacy among school-going adolescents in Botswana. Int Nurs Rev.

[10] Ezenwaka U, Mbachu C, Okeke C, Agu I, Ezumah N, Onwujekwe O (2021). Socio-demographic and economic determinants of awareness and use of contraceptives among adolescents in Ebonyi State, South-East. Afr J Reprod Health.

[11] Ezenwaka U, Mbachu C, Ezumah N, Eze I, Agu C, Agu I (2020). Exploring factors constraining utilization of contraceptive services among adolescents in Southeast Nigeria: an application of the socio-ecological model. BMC Public Health.

[12] Cleland J (2001). Illustrative questionnaire for interview-surveys with young people. Asking Young people about sexual and Reproductive behaviours. Illustrative Core instruments.

[13] Magadi M, Kaseje D, Wafula C, Kaseje M, Ochola-Odhiambo P, Ogutu-Owii S, et al. Sexual and reproductive health knowledge and behaviour of adolescent boys and girls aged 10–19 years in western Kenya: evidence from a cross-sectional pilot survey. J Biosoc Sci. 2021;1–12. 10.1017/S0021932021000353. 10.1017/S0021932021000353 34315560

[14] Phongluxa K, Langeslag G, Jat TR, Kounnavong S, Khan MA, Essink DR. Factors influencing sexual and reproductive health among adolescents in Lao PDR. Glob Health Action. 2020;13(sup2):28–37. 10.1080/16549716.2020.1791426. 10.1080/16549716.2020.1791426 PMC7480507 32741350

[15] Meena JK, Verma A, Kishore J, Ingle GK (2015). Sexual and Reproductive Health: knowledge, attitude, and perceptions among young unmarried male residents of Delhi. Int J Reprod Med.

[16] World Health Organisation. The changing world of adolescent sexual and reproductive health and rights. 2020. Available from: https://www.who.int/news/item/03-02-2020-the-changing-world-of-adolescent-sexual-and-reproductive-health-and-rights. Accessed Jan 2022.

[17] Rich LM, Kim SB (2002). Employment and the sexual and Reproductive Behavior of female adolescents. Perspect Sex Reprod Health.

[18] Kabiru C, Ezeh A (2007). Factors associated with sexual abstinence among adolescents in four sub-saharan African countries. Afr J Reprod Heal.

